# Poverty and food security: drivers of insecticide-treated mosquito net misuse in Malawi

**DOI:** 10.1186/s12936-019-2952-2

**Published:** 2019-09-18

**Authors:** Sara Berthe, Steven A. Harvey, Matthew Lynch, Hannah Koenker, Vincent Jumbe, Blessings Kaunda-Khangamwa, Don P. Mathanga

**Affiliations:** 1 PMI VectorWorks Project, Johns Hopkins Center for Communication Programs, Baltimore, MD USA; 2 Johns Hopkins Bloomberg School of Public Health, Department of International Health, Social and Behavioral Interventions Program, Baltimore, MD USA; 3 Department of Health Systems and Policy, College of Medicine, School of Public Health and Family Medicine, University of Malawi, Zomba, Malawi; 4 Malaria Alert Center, College of Medicine, University of Malawi, Blantyre, Malawi; 5 College of Medicine, University of Malawi, Blantyre, Malawi

**Keywords:** ITN, Misuse, Fishing, Lake Malawi, Malawi, Food security, Poverty

## Abstract

**Background:**

Over the past decade, food insecurity, connected to erratic rains and reduced agricultural outputs, has plagued Malawi. Many households are turning to fishing to seek additional sources of income and food. There is anecdotal evidence that insecticide-treated net (ITN) recipients in Malawi are using their nets for purposes other than sleeping, such as for fishing, protecting crops, and displaying merchandise, among others. The objective of this qualitative study was to explore the factors leading residents of waterside communities in Malawi to use ITNs for fishing.

**Methods:**

This study used qualitative and observational methods. Five waterside communities were identified, two each in the North, Central and Southern regions, representing a mix of lakeside and riverside settings. Fifteen focus group discussions were conducted with a total of 146 participants, including men, women, and community leaders.

**Results:**

Respondents stated that they knew that ITNs should be slept under to protect from malaria. Respondents discussed financial hardships their communities were facing due to droughts, poverty, food scarcity, unemployment, and devaluation of the Malawian currency, the *kwacha*. Many described selling household goods, including clothes and cooking pots, to generate short-term income for their family. Though no respondents admitted to selling an ITN themselves, the practice was commonly known. Participants said that food shortages were forcing them to make difficult choices. Fishing with ITNs was reported to be common in the study sites, as a response to food insecurity, and was widely understood to be harmful over the longer term. Respondents felt that it was everyone’s responsibility to cut down on this practice, but that efforts to confiscate or burn nets and boats of those caught fishing with ITNs were counter-productive since boats, especially, were a required resource for a productive livelihood. Respondents feared that if the health workers, government officials and donors continued to see ITNs being misused for fishing, the distribution of free ITNs would end, which would worsen malaria in their communities.

**Conclusions:**

Faced with economic hardships and food security crises, participants reported being forced to look for alternative incomes to feed their families. This sometimes included selling or repurposing their belongings, including ITNs, for income. This issue is complex and will require a community-led multisectoral response to preserve health, fisheries, and livelihoods.

## Background

Since 2004, national malaria control programmes and their implementing partners have distributed over one billion insecticide-treated bed nets (ITNs) for malaria prevention in sub-Saharan Africa [1]. ITN distribution and use has had a significant impact on malaria transmission during that time: WHO’s Global Malaria Programme estimates that deaths due to malaria have dropped from 607,000 to 435,000, a 28% decrease, between 2006 and 2016 [2].

In Malawi, ITNs remain the main vector control strategy [3]. However, despite cumulatively distributing approximately 27.5 million ITNs between 2004 and 2017 [1], malaria remains a significant problem. The country’s entire population remains at risk and transmission is reportedly increasing in some areas [4, 5].

Over the past decade, natural disasters and food insecurity, connected to erratic rains (potentially an effect of climate change [6]), have plagued the country. In Malawi, food insecurity is a threat to economic growth and long-term prosperity as well as the livelihoods of an already vulnerable population. Livelihoods have suffered as maize yields have dropped, leaving many families without enough food. Ninety per cent of the population is dependent on rain-fed agriculture, 60% of whom are food insecure on a year-round-basis [6]. Rain-fed agriculture is a major contributor to the national gross domestic earnings and supports 80% of Malawians’ livelihoods [7]. A volatile economy and chronic under-nutrition amongst children compound this issue. Given the economy and food insecurity, households are searching for additional sources of income to purchase the staples they once cultivated. Given the inherent need to feed oneself and one’s family, it is understandable that many are turning to fishing to provide either or both a source of income and food to stave off hunger in their households and malnutrition in their children.

With a downturn in the economy and resulting increase in food insecurity, and with the mass availability of ITNs for malaria prevention, there is both anecdotal and observational evidence that ITN recipients in Malawi are repurposing their nets for purposes other than sleeping, such as for fishing, protecting crops, and displaying merchandise, among many others. A recent Roll Back Malaria Partnership Consensus Statement of the Repurposing of ITNs, categorizes ITN repurposing in three ways: beneficial repurposing, neutral repurposing and misuse [8]. Beneficial repurposing still allows the user to be protected from malaria, including using older ITNs as window screens. Neutral repurposing does not prevent mosquito bites but its uses also cause no harm; examples include the covering of crops or using strips of old netting for string/rope. Misuse is clearly defined as any use of an ITN for purposes other than its intended use as a bed net to protect against malaria infection, with added environmental harm; fishing with an ITN is the prime example of misuse.

Though these anecdotal reports and observations of repurposing and misuse are widespread, there is little statistically valid and systematic data on the extent to which repurposing or misuse occur or the contexts in which they occur [9]. Small scale studies and surveys have found repurposing and misuse to be a problem, but quantifying the problem remains unknown. The key questions that remain to be answered include: how many, or what percentage, of the nets distributed are misused, and does misuse occur only after a the ITN has been deemed no longer effective for malaria prevention, or does it occur even if individuals and families remain unprotected? Recent studies conducted in the Democratic Republic of the Congo (DRC), Tanzania and Zambia, as well as global survey looking at the use of ITNs in fisheries begin to fill in some of these gaps. On Lake Tanganyika and on the Barotse floodplain, studies report that ITNs distributed through mass campaigns are sometimes used for fishing in waterside communities and that fish populations have decreased [10, 11]. The DRC study, carried out in an internally displaced persons camp, looked at economics as a driving factor of not using ITNs since a majority of ITNs were sold or exchanged, instead of being used for sleeping [12]. The global survey study received 94 independent observational inputs of the presence of ITNs being misused for fishing, primarily across sub-Saharan Africa and South and Southeast Asia [13]. Additionally, a recent publication positing the noncomplementary nature of four of the United Nations’ Sustainable Development Goals (SGD), suggests that in the context of receiving ITNs to protect oneself from malaria, the internationally community unknowingly provided a tool to end hunger and contradicted its efforts at ensuring sustainable ecosystems [14].

The objectives of this qualitative study were to document the extent to which residents of waterside communities in Malawi used ITNs for purposes other than sleeping, to catalogue the different types of repurposing, including misuse, and, to the extent possible, quantify the extent of the repurposing. The outcomes did not match the objectives, as explained later, due to the difficult nature of collecting relevant data to address quantifying the extent of the potential misuse. An exploration of the waterside observations and repurposed uses of ITNs will be the subject of a separate paper (Harvey et al. in preparation).

## Methods

### Study sites

**Fig. 1:**
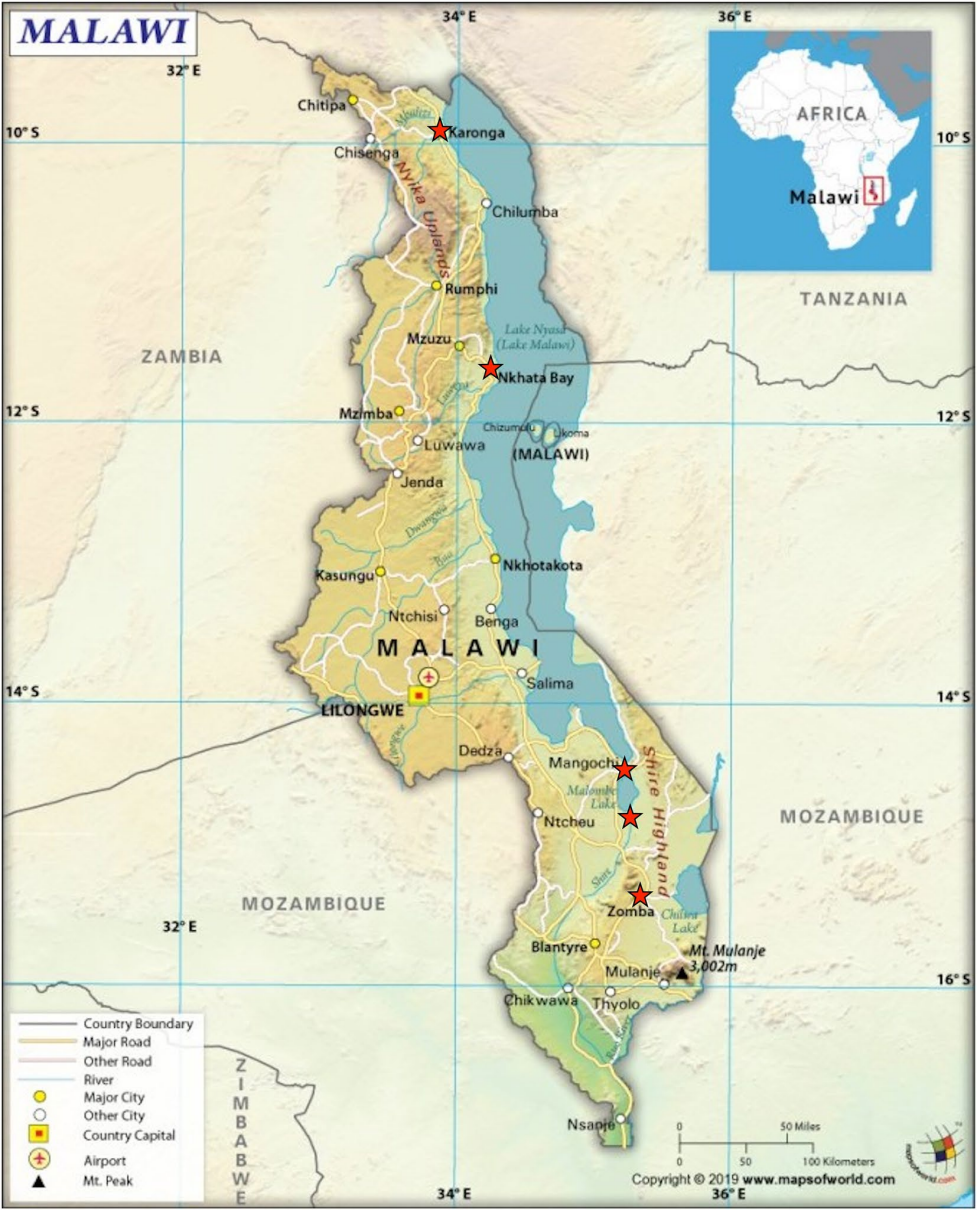
Map of Malawi with study sites marked in red

Data collection took place during March 2017, in five Malawian lakeside districts: Karonga and Nkhata Bay along the northwestern shore of Lake Malawi, Mangochi at the southern tip of the lake, Machinga along the Shire River, and Zomba on the western shore of Lake Chilwa. These communities represent varying cultural and geographic regions, different types of waterways and different modes of fishing (see Fig. 1). Additionally, the selected districts had high malaria transmission and high levels of ITN access (63%) [15]. It should be noted that no indoor residual spraying (IRS) took place in these study sites during the time of the study; the last IRS campaign that overlapped with these study sites was in Karonga in 2015 [3].

### Ethical approval

Ethical approval was obtained from the College of Medicine Research and Ethics Committee of the University of Malawi (Ref: P.11/16/2065) and Johns Hopkins University Bloomberg School of Public Health (Ref: IRB00007514). The study was administered only to participants above 18 years of age upon written informed consent.

### Data collection

**Fig. 2:**
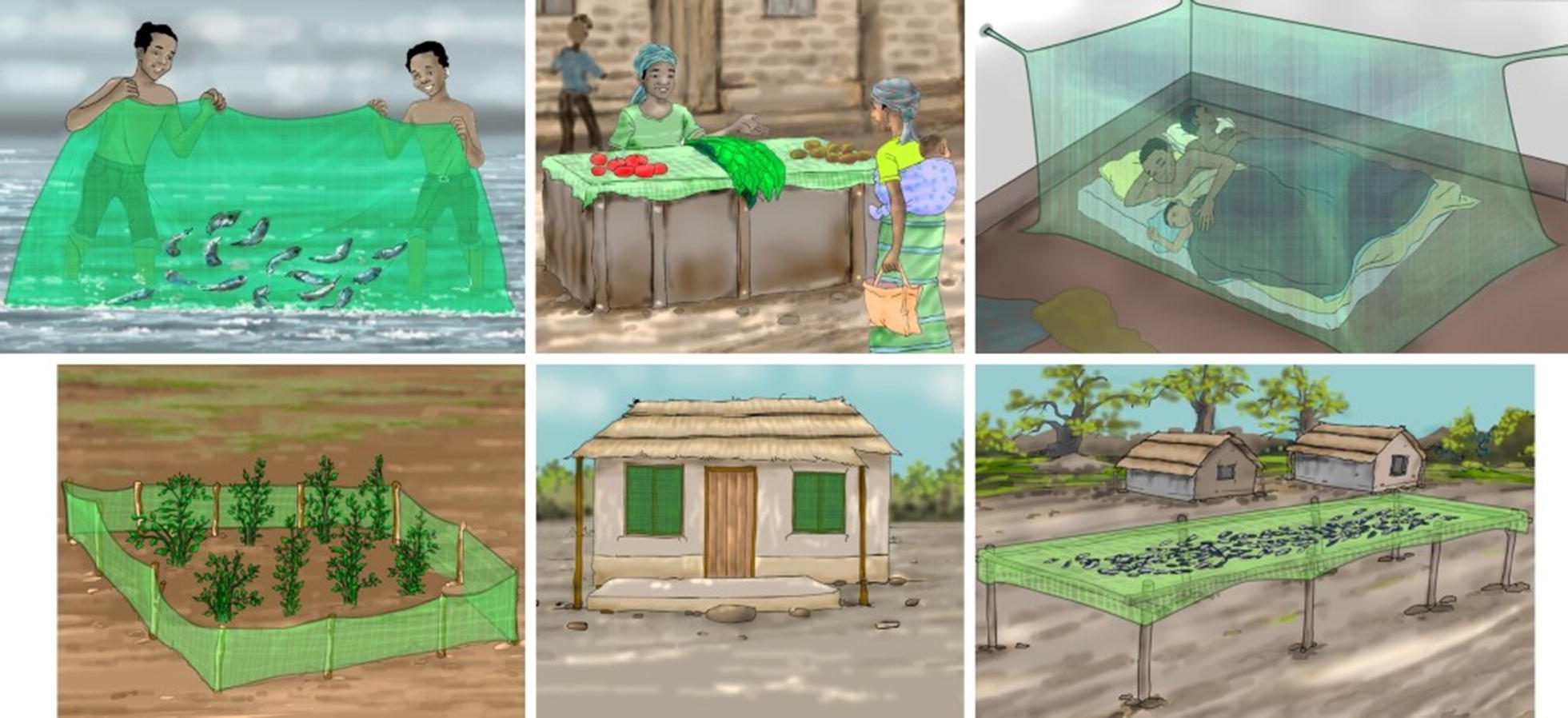
Illustrations of commonly observed ITN uses presented for discussion to focus group participants (note: original illustrations were all the size of an A4 sheet of paper [210 × 297 mm] they have been cropped here to enhance visibility in the article)

Three focus group discussions (FGDs) and observations were conducted in each of the five sites. Each FGD consisted of 10–12 participants: one amongst men, one amongst women and one amongst community leaders that could include a mix of men and women. At each site, all three FGDs took place simultaneously, so participation in one group precluded participation in any other. One facilitator, accompanied by one note-taker, led each FGD in one of five local languages. The facilitator followed a structured discussion guide developed around key topics of interest. As a part of the FGD, participants were asked to review and discuss their reactions to drawings illustrating the six most common ITN uses observed by two of the authors during an October 2016 formative research visit (see Fig. 2): (1) two men fishing with an ITN; (2) a market stand with produce displayed for sale on an ITN; (3) a family sleeping under an ITN; (4) ITNs used as a fence to protect crops; (5) ITN remnants used as window screens; and, (6) ITNs used as drying racks for fish. These drawings were produced by a local artist based on photographs taken in a few communities on the southwestern shore of Lake Malawi during the October 2016 visit.

FGDs lasted from 90 to 150 min and were audio recorded, transcribed into the local language, and translated into English throughout and following data collection. The study team debriefed each evening to discuss findings, make appropriate adaptations to the discussion guide, and review data quality.

### Data management and analysis

Three of the authors (SB, SAH, BKK) developed a preliminary codebook based upon FGD guides, research aims and ideas that emerged from the initial transcript reviews. This combination of deductive and inductive codes was tested on a sample of transcripts and then modified where needed to create a final codebook. All FGD transcripts were uploaded into ATLAS.ti, a qualitative data analysis software program, and coded using the final codebook. The same three authors then divided the FGD transcripts by participant type with SB coding the community leaders’ groups, SAH the men’s, and BKK the women’s. During coding, team members met periodically to compare findings and discuss interpretations of the data.

## Results

A total of 146 individuals participated in the 15 FGDs (male: n = 87; female: n = 52; unknown: n = 7). The majority of the FGD participants had lived in their respective communities for more than 10 years, with a few exceptions, such as teachers who had moved more recently. Although educational status varied, many did not complete primary school; few completed secondary. Participants’ self-reported occupations varied: while most were farmers or small business entrepreneurs, religious and community leaders, teachers, fishermen and members of forest and beach village committees were also represented. Many participants described malaria as a problem in their community and also acknowledged that ITNs were either used in the past or are currently being used in a variety of ways, including fishing. This acknowledgement validated the perception that ITN misuse for fishing is occurring in Lake Malawi, although it did not allow the researchers to make any reasonable estimate of the magnitude of the misuse.

### Economics or socio-economic environment in waterside communities

#### General changes from the past and insights into the future

Participants were asked for their perspectives on how things had changed in these communities from the past until present day. While some community members mentioned changes from as far back as 1965, most respondents referred to the past 5–15 years with their responses. Participants described both positive and negative changes, as seen in Table 1 below.

**Table 1:** General observations, changes in the community from past to present

Positive changes	Negative changes
Roads are more developed making transportation easier More/new health facilities are closer to their communities More/new schools which are closer to their communities Development in general is better, with family planning and safe motherhood initiatives	*Climate/environment* Erratic rains and soil erosion have negatively affected farming practices and crop yields Environmental degradation has increased: Trees are being cut for everything from fish drying racks to firewood and charcoal production. Cutting of trees also leads to erratic rains *Food security* Fish production in the lakes and rivers has decreased, negatively affecting livelihoods Food is scarce High population growth has strained resources leading to insufficient land for agricultural needs and overfishing *Health* Health outcomes are poorer, with more disease, not enough drugs (both quantity and effectiveness of current treatment), poor quality of care *Economics* Employment is scarce, especially amongst youth Devaluation/depreciation of the Kwacha

Given these perceptions of mostly negative changes from the past that cumulatively hurt livelihoods and negatively impact households, participants from this study did not see a bright future for their children or future generations. A male respondent from Machinga said,

“*The problem I see is that the environment is being destroyed. That is why I have also said that the future of children will be hard*.”

They recognized that overfishing of lakes and rivers will have detrimental consequences for future generations, that land is becoming scarcer and soil quality declining, and that the combination of these factors plus a growing population will lead to increasing food shortages. A female respondent from Karonga said,

“*Our children’s lives will be short. There will be no fish in the future for our children because mosquito nets catch a lot of juvenile and big fish*.”

In general, respondents believed that while the current situation is challenging, it was likely to continue to worsen in the future.

#### Food security and livelihoods

An increase in agricultural pests, specifically army worms, ruin crops before they can be harvested. Across the five sites, respondents said crop yields were down and families were facing major hurdles to feed themselves throughout the year. They reported that they typically had little worry immediately following the harvest, but that stocks tended to run out quickly. Participants in all sites stated that crop yields had decreased and mentioned that, for example, in the past, if a family harvested 10 bags of maize, they might eat 8 and sell 2. Now that same family would only harvest 5 bags of maize and would need to keep that to sustain their families. Male respondents from Nkhata Bay explained,

“*farming is no longer predictable nowadays. You may estimate that you will produce 15 bags only to find 10 or 6. So to sell and find some to eat it is not possible*.”

Another participant from Karonga described the food security problems:

*“There are some people who find it difficult to find food. Some people cultivate but there is not enough rainfall just like the way it is in our area, Ngara. We can say that all those who cultivated maize do not have food. The cassava that we have cultivated will be ready to be harvested next year as a result we will not have food this dry season, things won’t be fine. That’s the problem with food.”* (Male respondent, Karonga)

Participants said that they had to search for other means to feed themselves, typically through earning money to purchase goods at market. In these scenarios, multiple respondents reported turning to fishing (reportedly selling 90% of the catch), selling their possessions at a fraction of the cost they paid for them (even items as small as a used yard of fabric), or going as far as Mozambique or South Africa to find work.

As one woman from Karonga stated,

“*when you have shoes and you don’t have food at home, you sell it so that you buy food. Some people may have a mattress sell it to buy food, some sell pails or pots to buy food.”*

The majority of respondents throughout these sites eat *nsima* (thick corn meal) and occasionally rice with a limited variety of vegetables including pigeon peas, pumpkins, pumpkin leaves, and cassava. Many respondents reported that their families were food insecure and during the most difficult parts of the year (August–January, prior to harvest), that they and their children go to sleep hungry.

#### Malaria and ITN distribution

The most commonly reported health issues within the study communities were malaria and diarrhoea, followed by schistosomiasis, HIV/AIDS and cancer. While communities reported malaria as a health concern, there were differing opinions both within groups and across sites about whether participants’ homes and families were well protected from malaria. Some stated that malaria was an issue for their families, others that it had improved over the years or that they perceived the community to be well protected. Many participants reported that health facilities lacked personnel, medicines and equipment, adding to the challenge of dealing with any health concerns.

In all five study sites, participants stated that the most recent ITN distribution had occurred within the past year or two (2014–2015). Participants mentioned net distributions by partners such as Concern Universal and Population Services International as well as the government and health workers. Except in Mangochi, where participants reported having enough ITNs, responses differed about the adequacy of the ITN distribution. Most reported that a household registration had taken place and that families had been asked about the number of household members. Some thought that this number should have been verified by an external source. In any case, it was not clear to participants how the number of ITNs needed per household was calculated. Participants further reported that during distribution some families received one net per person, some received one for every two people, and some even fewer than one ITN for every two people. The following quote from Zomba illustrates the confusion among participants around the campaign’s quantification and distribution processes:

*“[Distribution] was dependent on your family size. If you have 7 children sometimes they could give you 5 nets. If you happen to have 4 children they could give you 3. If you have 1 child, they could give you 1 net. So it was dependent on your family size. If you happen to have 15 children as someone did say that he has 14 children, 10 mosquito nets would be given.”* (Male respondent, Zomba)

Respondents in the Machinga and Nkhata Bay FGDs added that during distribution, there were not enough ITNs, and those who showed up late to the distribution points did not receive nets. In the southern sites, especially along Lake Chilwa and in Mangochi, respondents described a practice of favouritism in net distribution. Several respondents mentioned this favouritism repeatedly. One participant from Machinga reported that when he sent his mother to collect his net on his behalf, his name was called but the village leader told the organizers not to give the net to his mother since he was well known to be a fisherman. This same respondent said that when his father went to the distribution site to exchange his own coupon for a net, someone had already come and claimed his net, leaving him empty-handed.

Across all of the study sites, participants recognized that challenges in net distribution were common and led to both under and over supplying households during mass campaigns. Many agreed with each other that village leaders should have verified the number of members in each household.

### Perceptions about correct and incorrect ITN use

The groups were then asked to discuss the advantages and disadvantages of using ITNs in each of the six scenarios illustrated in Fig. 2. Most participants stated that the family sleeping under the ITN was the only correct use and referred to the other scenarios as the incorrect use of an ITN. Many commented that the ITNs would better serve families if they were being used correctly. Some participants added that one would not use a new net in these scenarios, but one might use an old net once it was too worn to be effective for malaria prevention. Despite this, in most study sites participants acknowledged that the practices illustrated in the drawings were quite common. Based on their comments, participants were clearly aware that ITN use for fishing was illegal.

### The role of insecticide-treated nets in fishing

#### Using ITNs for fishing

On the whole, community members were open to discussing the issue of net misuse for fishing. While some were hesitant at first to broach the topic and used body language to discourage others from mentioning it, the data collectors assured participants that the study objective was to learn about the ways that ITNs are repurposed, not to penalize illegal or undesirable practices, and that the study team would protect their anonymity and confidentiality. In most groups, this was enough to overcome the hesitancy of most participants, though some remained suspicious of the study’s motives. Almost all FGD participants acknowledged that fishing with an ITN was an improper use and damaging to the present fish population.

As one participant from the community leaders FGD in Machinga said,

“*There is no future for our children there. We are killing the future of our children since what we are using is catching almost everything. If we left them, our children would have used fishing hooks and be able to catch fish. But, we are depleting all of that.”* (Community Leader, Machinga)

Several women from Machinga explained that when ITNs are used for fishing, the small mesh catches everything, including juveniles and fish eggs. This has negative consequences for both the environment and the fish supply. One woman stated,

“*It’s true, that way they are destroying the environment, mosquito nets catch all the fish. Big fish lay its eggs on the shores not in deep waters. So when fishing this way all the juvenile fish are captured by the net*.”

Another reiterated that fishing with mosquito nets

“*kill[s] juvenile fish that could have been killed in the future when the fish grows big.”*

There was less consensus about the long-term consequences. While many stated that the practice was already leading to or would eventually lead to the collapse of fisheries, others thought that fish populations would recover if there was more rain. Some thought that new species of fish would replace those that were disappearing.

FGD participants confirmed what had been anecdotally reported before the study: that many ITN recipients sell their nets to fishermen. Across all study sites, participants reported that one could sell a new ITN for 300–1000 Malawian kwacha (MK) (US$0.40–1.40) at the prevailing exchange rate during the study, depending on the location and the condition of the net. ITNs fetched less in sparsely populated northern lakeside communities and more in the more densely populated south. A lightly used ITN might yield MK 200–800 (US$0.30–1.10). Respondents stated that no one would purchase a heavily worn ITN with many holes, but noted that fishermen recovered such nets once they were discarded and used them to patch other fishing nets. Although none of the respondents reported selling their own ITN, many stated that selling ITNs was easy and could name places where buying and selling of nets took place. In a few other cases where participants knew of an ITN being sold, they stated that it was often children and adolescents who stole the ITN and sold it without their parents’ knowledge to have some money. In a few of these instances, participants mentioned that youth and men would sell the ITNs for money for beer.

In some of the sites, participants discussed that sometimes men walked through villages soliciting ITN sales. A male respondent from Machinga explained the process of buying and selling ITNs, following a mass campaign:

*“When the health personnel distribute the nets for malaria prevention the buyers come to coax people to sell their nets so that they should use them for fishing. They stopped buying where they sell the fishing gear because the mosquito nets are found in the community. They come and coax the people to sell, so because of poverty people sell to buy salt, maize flour. Because of poverty you do sell three or four and they use those nets for fishing.”* (Male respondent, Machinga)

In addition to describing the potential supply chain for the buying and selling of new ITNs, this respondent also showed how dire the economy has become wherein a person would sell their ITN for basic food staples. Participants from another site mentioned that following the mass ITN distribution, they knew of mass campaign workers selling ITNs at the dock directly to fishermen. As one participant in Zomba said,

“*We are not the only problem, those who distribute the nets are the ones who start selling the nets. They sell the nets to the fishermen. Instead of distributing the nets to people, they sell the nets*” (Female respondent, Zomba).

Some participants also stated that fishermen sometimes stood at the exit to health facilities and buy new nets from pregnant women who have just received them during an ANC visit. It is important to make clear that these are all reported behaviours; the study team did not directly observe any nets being sold or purchased.

### Enforcement/responsibility

When asked about enforcement or responsibility around stopping the ITN misuse for fishing, participants in all study sites responded that a solution to the issue is needed. Some stated that in the past, the fisheries department was patrolling the lakes and rivers and enforcing laws on illegal fishing and fishing gear, but that these efforts had slowed. In areas along Lake Malawi (Mangochi, Nkhata Bay, Karonga), the Department of Fisheries does still have a presence working in collaboration with community led co-management committees, or Beach Village Committees (BVCs). However, efforts to confiscate mosquito nets being used for fishing were not coordinated with other enforcement agencies (e.g., police). In the southern site around Liwonde, fishermen reported that officials from the nearby national park, within which a river flows, were confiscating and burning both nets and boats if found within the park. Fishermen and other community members described this as a serious threat to their livelihoods, and not as a solution. One fisherman reported losing several boats this way and expressed anger at the park rangers, and suspicion of study team members whom he assumed were working with the rangers, stating that no one seemed to care if his children went hungry.

A leader from Zomba describes his role in the BVC:

“*What we have been doing, since I am also part of a Beach Village Committee, our work is mainly to see to it that people don’t use mosquito nets for fishing. But there are many fishermen who are doing this now. We were able to stop this practice. The lake has demarcations we would go up to Chidambiya or Thongwe but they would tell us ‘our chiefs here allow us to use mosquito nets for fishing nets. Confiscate in your own area not here.’ We [BVC members] have a lot of work in stopping this practice. This is an ongoing program. Those who use mosquito nets are always doing it with great caution. BVC members go with the police and confiscate mosquito nets from the docks. They burn them because it is not acceptable.”* (Community Leader, Zomba*)*

Many participants stated that it was everyone’s responsibility to curtail the use of ITNs for fishing since overfishing affects everyone. In fact, FGD participants in two neighbouring communities accused one another of starting the practice of using mosquito nets for fishing; each stated independently they were only engaging in the practice themselves because if they did not, those from the neighbouring community would catch all the fish. Others proposed joint solutions between communities and government, using local leaders and traditional by-laws, and cited the successes of organizations like Ripple Africa in Nkhata Bay and the local BVCs. A participant from the community leader FGD in Nkhata Bay explained the role of Ripple Africa and their system of fines:

*“There is Ripple Africa which mainly helps with the issue of fish conservation. That is why we have already starting realizing that using mosquito nets [for fishing] is destructive. If one is found with a mosquito net [for fishing], [he] would be charged MK10,000. They also confiscate the materials. If the case is brought before court, [he] might pay another fine.”* (Community Leader, Nkhata Bay)

While fining those who use ITNs for fishing may not work in every case, it appears to be working for Nkhata Bay residents as participants from other study sites knew of Ripple Africa’s work and believed they were working to restore the environment. Men from both Mangochi and Karonga, respectively, believed that a multisectoral solution was the key. A majority of the respondents were worried that if fishing with ITNs continued and if the health workers, government officials and donors continued to see ITNs being misused for fishing, the distribution of free ITNs would end, which would worsen malaria in their communities.

*“The government, local leaders and the organizations should take a leading role of stopping the people because they have nothing to lose.”* (Male respondent, Mangochi)

*“For anything to happen, we need to join hands. The government has to take its part and the people as well. If the people can be empowered to take a leading role, the initiative can work.”* (Male respondent, Karonga)

## Discussion

This study reveals that a poor economy and food insecurity are leading drivers motivating some Malawians in the study sites to either use their ITNs for fishing or sell them for income. Families are struggling to feed themselves in an economically depressed time. The research proves Maslow’s Hierarchy of Needs wherein the basic physiological requirements of food, water and sleep, for example, are met before more complex needs such as safety, love and belonging, esteem and self-actualization [16]. Many Malawian families are hovering around the base of this pyramid to ensure that these basic needs, including physiological and safety, are met for their children and themselves. Unpredictable rains have exacerbated natural disasters and food insecurity, threatening economic growth and individual livelihoods [6]. Maize yields have declined and chronic under-nutrition continues to be a significant problem in the country [17]. Facing these challenges, households are unable to harvest sufficient staples and look for additional sources of income to purchase food. Fishing, sometimes with a readily available ITN, is an inappropriate, but understandable short-term solution. Respondents described the climate-related and agricultural challenges, including drought, nutrient-poor soil and rising costs incurred to maintain their farms. These issues, in conjunction with the availability of ITNs over the past several years through combined universal coverage campaigns and routine services (antenatal care and immunization clinics), are providing some families with a potential opportunity to sell their ITNs, but many will likely face the choice between protecting themselves from malaria or providing a meal for their children. In situations in which worn-out, older nets are being sold, the harmful impact on fish populations remains, but the risk of malaria infection can continue to be reduced through the use of a new ITN.

The opportunities to address the drivers of ITN misuse for fishing through malaria programming are very limited. Some improvements in managing household registration more tightly during campaigns are possible, and new vector control interventions, which might provide fewer opportunities for misuse may become more prevalent. However, it seems unlikely that there will be a large shift in malaria prevention technology away from ITNs in the next several years.

Nowhere in the study sites was it reported that individuals or families felt they had too many ITNs and that this was driving increased sales of ITNs for fishing. The available data on ITN access and use seems to suggest that ITNs are valued and used when people have access to them in their household [18]. However, Malawi’s strong ITN use rates among individuals with access does not preclude insufficient population ITN access as a result of repurposing, misusing, or selling ITNs. ITN durability monitoring might shed more light on ITN retention practices in these areas.

While ITN misuse for fishing is done to improve a household’s food availability in the immediate term, the practice exacerbates food insecurity and poverty over the medium and longer term. The most likely and direct source of harm from ITN misuse for fishing is through its impact on fish populations. The small mesh size of the nets means that juvenile fish of all species are caught, putting potentially serious pressure on the fishery. Reduced fish populations would result in reduced incomes for fishermen, and would also likely increase food insecurity in waterside communities. Given the findings that cash shortages and food insecurity are the main behavioral drivers for misuse of ITNs for fishing, this suggests that without action, a vicious cycle of increasing mosquito net fishing will increase the pressure to use ITNs for fishing.

Although opportunities to address the drivers of ITN misuse for fishing are limited within malaria programme activities, opportunities to address these drivers do exist in collaboration with the Ministry of Agriculture and its Department of Fisheries. Due to lack of proper tools and resources, data are lacking on fish populations and the distribution of species, limiting opportunities for mitigating measures such as establishing fish refugia and even identifying heavily used fishing areas in Lake Malawi. These data are essential for improved fisheries management practices to mitigate the pressure on fish populations, both from illegal use of ITNs and from legal fishing practices. It may also be possible to explore modifying legal fishing net sizes to better suit the consumer preferences for fish. There is significant consumer demand for smaller fish which are not caught with standard, large-mesh fishing nets, but are an important traditional food. Legalizing use of nets designed with a mesh size, which can capture these species (called *usipa* in Malawi and *dagaa* in Tanzania), without harming fish eggs and fish fry, might help reduce incentives to employ mosquito nets for fishing. Thus, increased investment in fisheries management is critical for data collection, monitoring and mitigating the decline of the fishery.

While data-backed evidence from other countries is limited, recent small-scale studies in the DRC, Tanzania and Zambia have produced similar findings. In an internally displaced persons camp in DRC, participants were often selling their ITNs due to food insecurity [12]. In Tanzania, a study along the shore of Lake Tanganyika reported 46.8% of survey respondents reported using their ITN for fishing for income; 38% had done so due to hunger [10]. Many of these participants were aware that ITN use for fishing was illegal. Though the Zambia case did not specifically investigate drivers of ITN use for fishing, it found widespread misuse of ITNs for fishing in Western Zambia and recommended a multi-sectoral approach moving forward [11]. A recent global survey found both push (the ease of which ITNs are available and convenience of use) and pull (poverty, lack of alternative food sources) factors as the main drivers behind this behavior; these pull factors echo the drivers from this study [13]. It found as well that a cluster of these observations originated from the African Great Lakes, of which Lake Malawi is included.

Given the complexity of the issues surrounding ITN misuse for fishing, solutions will require a multisectoral approach. While malaria programmes can and should play a role, specifically in optimizing malaria prevention and integrated vector control management, livelihoods, fisheries, food security and enforcement agencies must also be involved. The recent conflicting SDG publication suggests that these cross-sectoral opportunities are the key to any successful way forward; single sector approaches are incapable of weighing the pros and cons of different interventions [14]. Livelihoods experts should continue to support existing or new programmes that women and families can engage into improve both subsistence livelihoods and income generation. Although the data are incomplete or outdated, it seems that the fish populations in Lake Malawi are declining [19], thus fisheries experts are needed to mitigate the overfishing problems and work with local enforcement officials to effectively conserve and restore the existing fish population. Critical stakeholders such as community leaders, women’s group leaders and fishermen should also be included in discussions and planning ways to increase community engagement with the problem. To date, enforcement has been under-effective. Community members across the five sites believed that it was “everyone’s responsibility” to curtail the use of ITNs for fishing; unfortunately, this common responsibility often became no one’s responsibility and most communities have not intervened. Before effective enforcement can be done, the individuals, groups or communities using ITNs for fishing must take responsibility. Once they have, Beach Village Committees may be a viable option to monitor their own communities. Ripple Africa has developed a useful guide for communities to discourage ITN use for fishing and encourage conversation efforts [20].

### Study limitations

This study had several limitations. Although the sampling sought to collect data from various waterside communities, the findings do not reflect the attitudes and actions of each of their communities nor of all waterside populations in Malawi. They also do not reflect the national attitudes and actions. As noted above, this study encountered situations of social desirability bias; the study respondents knew that certain actions, like fishing with mosquito nets or selling them, were disapproved of and therefore did not always want to provide details of these actions or admit to partaking in the activities. Recognizing this potential for biased responses, the data collectors continually reminded respondents that their comments were anonymous and that they should be as open and honest as possible.

## Conclusions

Economic hardships and food security crises were cited as the key drivers of selling ITNs and using ITNs for fishing in the study sites. This research reinforced prior anecdotal evidence suggesting that ITNs were misused for fishing in waterside communities in Malawi. Families feel forced to choose between protecting their family from malaria or feeding them a meal. The misuse of ITNs for fishing is a complex problem in Malawi and an effective response will require a multisectoral collaboration, including community ownership of solutions, and investment and guidance by food security, malaria, enforcement, climate change and fisheries experts.

## Data Availability

The qualitative transcripts and codebook reports used and/or analyzed during the current study are available from the corresponding author on reasonable request.

## Abbreviations

BVC: Beach Village Committee
DRC: Democratic Republic of the Congo
FGD: focus group discussion
HIV/AIDS: human immunodeficiency virus/acquired immune deficiency syndrome
ITN: insecticide-treated net
MIS: Malaria Indicator Survey
NGO: non-governmental organization
PMI: President’s Malaria Initiative
WHO: World Health Organization
